# Dilated Virchow–Robin Space and Cognitive Impairment in People With Parkinson’s Disease: A Systematic Review

**DOI:** 10.1155/rrp/6959099

**Published:** 2026-11-06

**Authors:** Mehri Salari, Maryam Mirabedini, Kamran Rezaei, Masoud Etemadifar

**Affiliations:** ^1^ Men’s Health and Reproductive Health Research Center Shahid Beheshti University of Medical Sciences Tehran Iran sbmu.ac.ir; ^2^ Faculty of Medicine Shahid Beheshti University of Medical Sciences Tehran Iran sbmu.ac.ir; ^3^ Faculty of Medicine Isfahan University of Medical Sciences Isfahan Iran mui.ac.ir

**Keywords:** cognition, glymphatic, Parkinson’s disease, perivascular space, Virchow–Robin space

## Abstract

Virchow–Robin space (VRS) is characterized by small cavitary or cystic structures in the brain that represent morphological markers of altered interstitial fluid dynamics within the perivascular (glymphatic) pathway, originating from small vessels in the subarachnoid space and penetrating the brain. Their dilation has been associated with altered clinical status in Parkinson’s disease (PD). The present study aims to review the reported relations between dilated VRS (dVRS) and cognitive impairment in patients with PD. A total of 71 articles were identified through database searching, and after reviewing based on the eligibility criteria, 16 studies were selected. Across the directly relevant PD studies, dVRS burden was associated with lower scores on some cognitive tests in some, but not all, cohorts; several studies reported null or only indirect associations. The most commonly used assessment tool was Montreal Cognitive Assessment (MoCA); the Mini‐Mental State Examination (MMSE), Frontal Assessment Battery (FAB), Seoul Neuropsychological Screening Battery (SNSB), and Parkinson Neuropsychometric Dementia Assessment (PANDA) were also used across the included studies. Overall, the presence of the dVRS in basal ganglia in patients with PD was frequently associated with the vascular risk factors, and the resulting mechanical mass effect (local compression and displacement of adjacent neural structures by the dilated, fluid‐filled spaces), impaired waste clearance, and vascular impact have been proposed as pathways potentially relating dVRS to cognitive status in these patients. Cognitive decline in the case of basal ganglia dVRS is shown to be associated with crossing inferior fronto‐occipital and inferior longitudinal fasciculus fibers.

## 1. Introduction

Virchow–Robin space (VRS) is characterized by small cavitary or cystic structures in the brain related to the perivascular space surrounding small vessels in the subarachnoid space penetrating the brain as the glymphatic system [1, 2]. These spaces contain a mixture of cerebrospinal fluid (CSF) and interstitial fluid (ISF) and form part of the perivascular CSF–ISF exchange pathway underlying glymphatic clearance; they therefore appear isointense to CSF on T2‐weighted magnetic resonance imaging (MRI) [3, 4]. Most VRS are small and asymptomatic. When markedly enlarged, they are termed dilated Virchow‐Robin spaces (dVRS) and may disrupt neural circuitry through impaired glymphatic clearance of metabolic waste and misfolded protein species [1, 5, 6].

Parkinson’s disease (PD) is a neurodegenerative disorder capable of exhibiting motor—tremor, bradykinesia, and rigidity—and nonmotor symptoms; and cognitive decline in domains such as memory, language, and perception is an important part of nonmotor symptoms [4, 6–8]. The pathophysiology of PD consists of multiple components. One of these is associated with increased aggregation of protein components called Lewy bodies in various parts of the brain, including basal ganglia, midbrain, spinal cord, and cortex [6]. Impaired glymphatic clearance of α‐synuclein monomers and oligomers facilitates their aggregation into Lewy bodies; dilated perivascular spaces are a structural correlate of this clearance failure on MRI [3].

There is a regional predilection for dVRS in the basal ganglia, cortex, and brain stem of patients with PD. The basal ganglia predominance is mechanistically plausible: the lenticulostriate arteries arise directly from the middle cerebral artery and are therefore exposed to higher pulsatile arterial pressure and reduced damping than cortical arterioles, while the high metabolic demand of the basal ganglia generates a correspondingly high interstitial‐solute load. Together, these features make the basal ganglia perivascular network particularly susceptible to perivascular stasis, glymphatic clearance failure, and subsequent dilation [9, 10]. This structure can present as a single unilateral perivascular space, or as much as hundreds of these structures distributed bilaterally in each of the mentioned sites [1, 2, 4, 5]. Age, hypertension, and the presence of white‐matter lesions in the MRI of PD patients are defined as risk factors for dVRS in the basal ganglia [5]. The anatomical proximity of dVRS structures to basal ganglia components in patients with PD led some researchers to study if there is any relation between dVRS and clinical manifestations in PD, consisting of not only the basal ganglia‐related motor symptoms but also the cognitive decline [4].

The present study aims to systematically review the reported relationships between dVRS and cognitive impairment in patients with PD, and to synthesize the proposed underlying mechanisms (impaired glymphatic waste clearance, mechanical mass effect on adjacent neural structures, and disruption of cognition‐related white‐matter tracts).

## 2. Methods

### 2.1. Design

This systematic review was conducted based on guidelines provided in Preferred Reporting Items for Systematic Reviews and Meta‐Analyses (PRISMA) [11].

### 2.2. Search Strategy

PubMed, Scopus, and Web of Science databases were searched to identify relevant articles (January 1, 2000, to July 1, 2026). Reference lists of all included studies and three relevant systematic reviews were hand‐searched for additional eligible articles. No language restriction was applied at the database level; non‐English records were excluded at full‐text screening. The search strategy consisted of entry words and phrases including (“virchow robin^∗^” OR “virchow‐robin”) AND (parkinson^∗^) AND (cognit × OR metacognit × OR awareness OR comprehens × OR conscious × OR intuit × OR imagin^∗^), which resulted in a total number of 71 articles. The search excluded grey literature, conference proceedings, and dissertations. The full database‐specific search strings and dates for PubMed, Scopus, and Web of Science are provided in Supporting Table S2.

### 2.3. Eligibility Criteria

Original articles published in the English language reporting the cognitive impairment in human subjects with PD and presence of dVRS in their brain imaging were considered suitable for this study (Figure 1). Studies were classified a priori into two groups. Primary studies directly evaluated the association between dVRS (or MRI‐visible enlarged perivascular‐space burden) and cognition in a PD cohort. Contextual studies informed mechanism or risk factors but did not directly evaluate a dVRS–cognition association in a PD cohort (e.g., cohorts without PD participants, without a cognitive outcome, mixed non‐PD populations, or biomechanical models); these were retained for interpretive context and are reported separately throughout.

**FIGURE 1:**
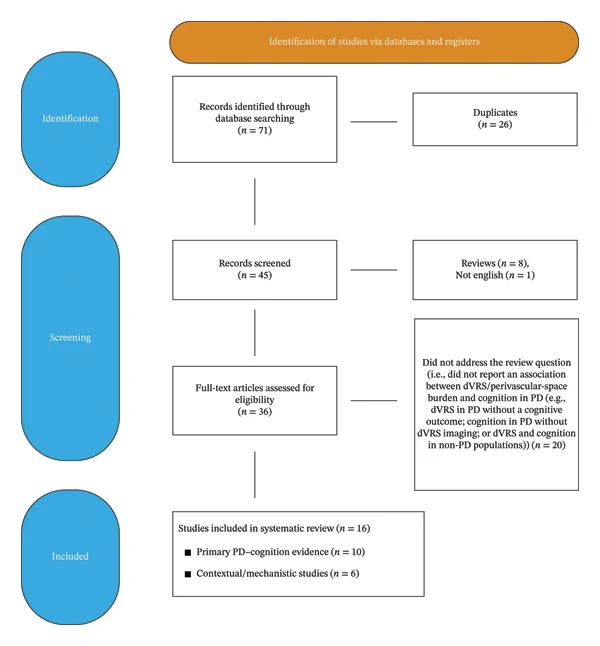
PRISMA flow diagram depicting the flow of information through the different phases of a systematic review. Exclusions at the full‐text stage were based on relevance to the review question, not on the direction or statistical significance of study findings.

### 2.4. Study Selection and Data Extraction

Data were extracted independently and in duplicate by two reviewers (K.R. and M.M.) using a prepiloted, standardized extraction form. Extracted fields comprised study design and country; sample size (PD and comparator); PD diagnostic criteria and severity (Hoehn & Yahr/MDS‐UPDRS); MRI field strength and sequences; dVRS rating method (visual ordinal scale, semiquantitative count, or automated volume‐fraction quantification) and anatomical regions assessed; cognitive instruments; the principal effect estimates with 95% confidence intervals where reported; and the confounders adjusted for. The form was piloted on three studies before full extraction. Discrepancies were resolved by discussion, with a senior reviewer (M.S.) adjudicating any residual disagreement; agreement was documented for each study.

### 2.5. Risk of Bias Assessment

Risk of bias was assessed independently and in duplicate (K.R. and M.M.), applying a design‐appropriate Joanna Briggs Institute (JBI) Critical Appraisal Checklist to every included study: the JBI checklist for Analytical Cross‐Sectional Studies for the 10 analytic (cross‐sectional, case–control, and cohort) studies; the JBI checklist for Case Series for the 3 case series; and the JBI checklist for Case Reports for the 3 case reports. Each item was rated Yes/No/Unclear/Not applicable; an overall judgment (low, moderate, or high risk of bias) was assigned per study. Disagreements were resolved by discussion with a senior reviewer (M.S.). Per‐item and overall ratings for all 16 studies, grouped by design and evidence tier, are provided in Supporting Table S1.

## 3. Results

### 3.1. Study Selection

Database searching identified 71 records. After removal of 26 duplicates, 45 records were screened by title and abstract. Of these, nine were excluded (eight reviews and one non–English‐language article). The remaining 36 full‐text articles were assessed for eligibility, and 20 were excluded because they did not report on the association between VRS and cognitive status in PD (e.g., they reported VRS in PD without a cognitive outcome, or cognition in PD without VRS imaging, or VRS and cognition in non‐PD populations). Exclusions at full‐text stage were based on relevance to the review question, not on the direction or significance of the findings. Sixteen studies were included in the final review (Figure 1). Throughout this review, negative correlations between dVRS burden and cognitive test scores indicate worse cognitive performance with greater dVRS burden.

### 3.2. Study Characteristics

Of the 16 included studies, 10 were classified as primary evidence directly evaluating the dVRS–cognition association in PD (7 analytic studies and 3 PD case reports), and 6 as contextual/mechanistic literature (Table 1). Findings are reported below and in the Discussion under these two headings, and the risk‐of‐bias assessment and Table 1 are grouped accordingly. Designs comprised five cross‐sectional studies, one longitudinal cohort, four case–control studies, three case series, and three case reports. Studies originated from China (*n* = 5), the USA (*n* = 4), and Korea (*n* = 3), with one each from Spain, Italy, Sweden and Latvia. The seven group 1 analytic studies contributed 650 patients with PD. Three studies (Fang et al. [12], Donahue et al. [6], and Li et al. [18]) drew on the Parkinson’s Progression Markers Initiative (PPMI) cohort, so partial participant overlap between them cannot be excluded.

**TABLE 1:** Characteristics and principal findings of the included studies, grouped by evidence.

Study (ref), country	Design; *n* (PD/comparator)	MRI field; dVRS method (reliability)	Regions assessed	Cognitive measure	Key finding relevant to dVRS–cognition	Direction of finding
*Group 1—Primary evidence: direct dVRS–cognition studies in PD*						
Donahue 2024 [4], USA	Cross‐sectional; 50 PD (H&*Y* ≤ 3)/none	3.0T; automated PVS volume fraction	CSO; prefrontal WM; BG	MoCA + 5‐Domain composite	Rostral‐middle‐frontal PVS with lower MoCA: r (48) = −0.524, *p* < 0.001; adjusted *β* = −318.40 (95% CI −613.85 to −22.95), *p* = 0.047. BG PVS with lower global cognition: *β* = −84.65 (−147.55 to −21.76), *p* = 0.030	Supportive (adjusted)
Tu 2022 [7], China	Prospective cross‐sectional; 26 PD (+19 VaP)/32 HC; H&Y 3.0	3.0T; visual count on T2WI (Adams criteria)	BG, CSO, brainstem, hippocampus	MoCA‐Beijing	Within‐PD, EPVS number, and location did not differ between PD‐CI and PD‐NCI (all *p* > 0.05). Significant BG‐EPVS associations were confined to the vascular parkinsonism group	Null (adjusted)
Fang 2020 [12], China (PPMI)	Longitudinal cohort; 245 PD (drug‐naïve, H&Y 1–2)/98 HC	1.5T or 3T; Potter 4‐point visual scale, 2 blinded raters (*κ* BG 0.75)	CSO, BG, midbrain	MoCA, HVLT, BJLOT, LNS, Semantic Fluency, SDM	No association with global cognition, memory, working memory, or executive function (MoCA vs. BG‐EPVS *β* = 0.06 [−0.50 to 0.62], *p* = 0.825). High BG‐EPVS with worse attention–processing speed (SDM *β* = −2.03, *p* = 0.050)	Mixed, largely null (adjusted)
Shin 2021 [13], Korea	Cross‐sectional registry; 154 PD (36 intact/111 MCI/7 PDD)	3.0T; validated 5‐point visual scale (STRIVE), blinded (*κ* BG 0.74)	BG, white matter	SNSB	Association with cognition ran through WMH rather than directly: BG‐dPVS to total WMH *β* = 0.440 (SE 0.079), *p* < 0.001; total WMH to cognition *β* = 0.188 (SE 0.044), *p* = 0.047 (path analysis)	Indirect, WMH‐mediated (adjusted)
Shen 2021 [14], China	Cross‐sectional; 40 early‐stage PD (H&Y 1–2.5)/41 HC	7T; manual count + volume (ITK‐SNAP), single reader (ICC BG 0.81)	BG, midbrain	MMSE, MoCA, PANDA	PVS burden not correlated with MMSE, MoCA, or PANDA. BG PVS count correlated with motor severity (MDS‐UPDRS) and LEDD only	Null (adjusted)
Kim 2007 [1], Korea	Case report; 1 (67 y, M)	Field NR; qualitative description	Periventricular & subcortical WM	K‐MMSE 10/30; CDR 1; GDS 3	Polycystic dVRS with dementia and parkinsonism; co‐occurrence only	Descriptive (single case)
Shoeibi & Litvan 2017 [15], USA	Case report; 1 (28 y, M)	Field NR; qualitative (giant VRS > 1.5 cm)	Posterior fossa (midbrain, thalamus, pons)	MoCA 25/30	Mild executive dysfunction (impaired retrieval, reduced fluency) with giant VRS; co‐occurrence only	Descriptive (single case)
López Fernández 2016 [16], Spain	Case report; 1 (55 y, M)	Field NR; qualitative description	Bilateral BG, internal capsules, frontoparietal periventricular WM	MMSE 28/30; Fototest 32	Mild delayed memory and executive impairment with parkinsonism; co‐occurrence only	Descriptive (single case)
Stagisa 2026 [17], Latvia	Cross‐sectional; 21 PD/21 HC	3.0T; count + Potter/Heier rating scales	BG, CSO	MoCA	Largest per‐slice PVS counts with lower MoCA: CSO right *ρ* = −0.421, *p* = 0.003; CSO left *ρ* = −0.431, *p* = 0.002; right BG *ρ* = −0.362, *p* = 0.012	Supportive (unadjusted; small sample)
Li 2025 [18], China (PPMI)	Case–control; 114 de novo PD/55 HC	Field NR (PPMI); EPVS burden rating	CSO, BG	Cognitive + motor batteries; CSF GFAP	CSO EPVS with worse cognition; BG‐EPVS with motor impairment only. EPVS‐to‐symptom mediation via GFAP nonsignificant	Mixed, region‐dependent (unadjusted correlation + mediation)

*Group 2—Contextual, mechanistic, or supporting studies (not direct dVRS–cognition evidence in PD)*						
Zhang 2016 [5], China	Case–control (lacunar‐stroke cohort; no PD); 89 severe BG‐EPVS/178 controls	Field NR; visual rating 0–4 on T2WI	BG, CSO	Not measured	Independent risk factors for severe BG‐EPVS: brain atrophy OR 1.40 (1.13–1.73); deep WMH OR 1.88 (1.24–2.83)	No cognitive outcome
Donahue 2021 [6], USA (PPMI)	Case–control; 246 PD (179 iPD + 67 fPD)/185 comparators (101 NMC + 84 HC)	3.0T; automated PVS volume fraction	Global and regional cortical/subcortical WM	Not the primary outcome	PVS volume fraction higher in PD than non‐PD (*F* = 5.4, *p* = 0.021); superior temporal *F* = 9.6, *p* = 0.0021; medial orbitofrontal *F* = 6.0, *p* = 0.014	Burden comparison only; no cognitive outcome
Burnett 2014 [8], USA	Retrospective case–control; mixed elderly (∼19% parkinsonian): 27 severe SCS/52 controls	Field NR; blinded visual review (Swiss cheese striatum)	Striatum	MCI/dementia by medical‐record review	No difference vs. controls: MCI 15% vs. 8% (*p* = 0.44); dementia 30% vs. 21%, OR 1.57 (0.48–5.13); parkinsonism 19% vs. 17%. SCS correlated with leukoaraiosis (*p* < 0.001)	Null (not PD‐specific)
Scollato 2016 [2], Italy	Retrospective case series; 7 (mean 78.7 y); not PD‐specific	T2 (field NR); count (> 20 eVRS), Evans ratio < 0.30	BG and periventricular parenchyma	Cognitive disturbance (NPH‐like; not formally scored)	eVRS without ventriculomegaly improved after CSF diversion (PELD ± VP shunt); supports a CSF‐stasis mechanism	Mechanistic (no PD‐specific cognitive test)
Alonso 2021 [19], Sweden	Computational case series (DBS modelling); 7 PD	MRI (field NR); VRS modeled as high‐conductivity spheres	Subthalamic nucleus	Not assessed	VRS within or near the STN deform the DBS electric field (FEM simulation); ∼10% of DBS patients had VRS	Not applicable (biomechanical)
Lee 2015 [20], Korea	Retrospective case series; 11 parkinsonism (only 4 PD; 3 VaP, 2 MSA, 1 PSP, 1 FTD)	1.5T; semiquantitative (0–3) + quantitative count (ICC 0.964)	Striatum	K‐MMSE, MoCA‐K, FAB	dVRS score vs. FAB *ρ* = −0.868 (*p* < 0.01); vs. MoCA‐K *ρ* = −0.847 (*p* < 0.01); vs. K‐MMSE *ρ* = −0.675 (*p* < 0.05)	Supportive but heterogeneous (unadjusted; only 4/11 PD)

*Note:* Direction is coded with respect to the hypothesis that greater dVRS burden accompanies worse cognition; “supportive” therefore corresponds to negative correlations with cognitive test scores. “Adjusted” indicates that the reported estimate derives from a multivariable model; the full covariate list for each study is given in Supporting Table S1. CSO, centrum semiovale; BJLOT, Benton Judgment of Line Orientation; FTD, frontotemporal dementia; iPD/fPD, idiopathic/familial PD; MoCA, Montreal Cognitive Assessment; PNDA, Parkinson Neuropsychometric Dementia Assessment; LED/LEDD, levodopa‐equivalent (daily) dose; STN, subthalamic nucleus; STRIVE, Standards for Reporting Vascular Changes on Neuroimaging; VaP, vascular parkinsonism; (d/e)PVS, (dilated/enlarged) perivascular spaces; VP, ventriculoperitoneal.

Abbreviations: BG, basal ganglia; CDR, Clinical Dementia Rating; DBS, deep brain stimulation; FAB, Frontal Assessment Battery; FEM, finite‐element model; GDS, Global Deterioration Scale; GFAP, glial fibrillary acidic protein; HC, healthy control; HVLT, Hopkins Verbal Learning Test; H&Y, Hoehn &Yahr; LNS, Letter‐Number Sequencing; MMSE, Mini‐Mental State Examination; MSA, multiple system atrophy; NMC, nonmanifesting carrier; NPH, normal‐pressure hydrocephalus; NR, not reported; PD, Parkinson’s disease; PELD, percutaneous endoscopic lumbar discectomy; PPMI, Parkinson’s Progression Markers Initiative; PSP, progressive supranuclear palsy; SCS, Swiss cheese striatum; SDM, Symbol Digit Modalities; SNSB, Seoul Neuropsychological Screening Battery; VRS, Virchow–Robin space; WMH, white‐matter hyperintensities; WM, white matter.

Hoehn & Yahr stage was reported in four studies [4, 7, 12, 14]. Shin et al. [13] instead stratified by cognitive stage, and Fang et al. [12] and Li et al. [18] characterized their cohorts as drug‐naïve or de novo; the remaining studies did not report motor severity.

MRI field strength was 3.0T in five studies, 7T in one [14], 1.5T in one [20], and mixed 1.5T/3.0T in one [12]; field strength was not reported in the remaining eight, including all three case reports. dVRS ascertainment used automated volumetric quantification in two studies (both by Donahue et al. [4, 6]), a published ordinal visual rating scale in six (Potter, Heier, or STRIVE‐consistent 0–4 equivalents [5, 12, 13, 17, 20]), count‐based enumeration in two [7], combined manual count with volumetry in one [14], and qualitative identification without a formal rating instrument in five (the three case reports, together with [8, 19]). Studies using more than one approach were assigned to their primary method; interrater reliability was reported in only four studies (*κ* 0.74–0.75 for basal ganglia rating [12, 13]; ICC 0.81 and 0.964 [14, 20]).

The principal cognitive instruments were the Montreal Cognitive Assessment (MoCA) (*n* = 7), Mini‐Mental State Examination (MMSE) (*n* = 4), Seoul Neuropsychological Screening Battery (SNSB) (*n* = 1), FAB (*n* = 1), and Parkinson Neuropsychometric Dementia Assessment (PANDA) (*n* = 1); several studies used more than one instrument. Cognition was not assessed in three contextual studies [5, 6, 8, 19], was described but not formally scored in one [2], and was ascertained retrospectively from medical records in one [8]. Detailed characteristics for each study are presented in Table 1.

### 3.3. Risk of Bias Assessment

All 16 studies were appraised (Supporting Tables S1). Risk of bias was low to moderate for the analytic (cross‐sectional, case–control, and cohort) studies, and moderate to moderate‐high for the case series and case reports, reflecting their uncontrolled designs and small, sometimes clinically heterogeneous, samples.

### 3.4. VRS Sites in Patients With Parkinson’s Disease

The sites where the dVRS are developed are potentially related to the clinical manifestation in PD patients. The dVRS may present in both younger and elderly population; however, these structures are less severe in terms of clinical manifestation in the elderly populations [2, 5]. The dVRS structures—generally referred to as perivascular spaces—are reported in PD patients with degrees of cognitive decline in various sites of the brain, including midbrain, subcortical white matter, thalamus [6, 7], cortex [6], globus pallidus, putamen, substantia nigra, and subthalamic nucleus [19, 20]. Thus, cortical and subcortical white‐matter regions for their functional role in cognitive status, and basal ganglia and brain stem regions due to passage of specific nerve bundles (visualized on diffusion tensor imaging (DTI) and tractography, e.g., inferior fronto‐occipital fasciculus (IFOF) and inferior longitudinal fasciculus (ILF)) are considered potential places where the presence of dVRS may be responsible for cognitive decline in PD patients (Figure 2) [7].

**FIGURE 2:**
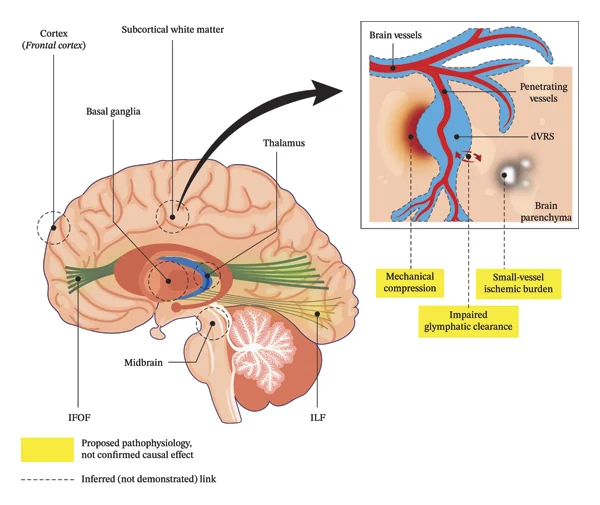
Proposed pathophysiological model linking dilated Virchow–Robin space (dVRS) to cognitive decline in patients with Parkinson’s disease (PD). Coronal/sagittal schematic of the brain shows reported anatomical sites of dVRS in PD‐related cognitive decline (frontal cortex, subcortical white matter, basal ganglia, thalamus, midbrain). The cognition‐related white‐matter fiber tracts shown are the inferior fronto‐occipital fasciculus (IFOF) and the inferior longitudinal fasciculus (ILF). The upper‐right inset is a hypothesis‐generating schematic of the three proposed downstream mechanisms (proposed mechanical compression of adjacent neural structures, proposed impaired glymphatic clearance, and proposed small‐vessel ischemic burden) linked to the dVRS by dashed inferred‐relationship arrows. Mechanisms shown are consistent with current evidence but have not been individually validated in directly comparable PD cohorts. dVRS: dilated Virchow–Robin space, IFOF: inferior fronto‐occipital fasciculus, ILF: inferior longitudinal fasciculus.

When notably enlarged, the VRS in the striatum is considered Swiss cheese striatum (SCS) and is considered a potential risk for increased cognitive decline in PD patients [8]. Concerning the role of cortical region in high‐function cognitive status, a study conducted by Donahue et al. on 246 PD patients and 185 controls reported that the cortical and subcortical white‐matter VRS volume fraction (total volume of VRS in the cortex or subcortical white matter over total volume of the cortex of subcortical white matter) was significantly higher in PD patients compared to the controls (*F* = 5.4, *p* = 0.021), and in terms of regional comparison in the cortical and subcortical white‐matter region, the VRS in PD patients had significantly more volume fraction in the superior temporal region and the medial orbitofrontal region [6]. Although the dVRS affects the site where it is developed in many ways, a study conducted by Tu et al. reported that the location of dVRS was not significantly different in vascular parkinsonism patients with and without cognitive impairment [7].

### 3.5. Effects on MoCA

Most of the studies in the literature considered MoCA as the assessment tool for cognition status in the setting of the presence of dVRS in PD patients (Table 1) [20]. Donahue et al. conducted a study on 50 PD patients to determine whether the volume fraction of VRS (i.e., the volume of the region of interest that is composed of VRS structures) is significantly related to the PD cognitive clinical manifestations [4]. They defined the cognition level as five domains: executive function, memory, visuospatial function, attention, and language. In addition, they assessed global cognition as the mean of the mentioned five domains’ z‐scores, and MoCA score as the general cognition screen. Following the linear regression for covariates, they reported that the rostral‐middle‐frontal dVRS had a significant negative correlation with visuospatial function, MoCA, and global cognition z‐score [4]. They also reported that basal ganglia dVRS volume fraction was negatively correlated with MoCA and global cognition z‐score [4]. It is important to note that the effect of frontal lobe perivascular‐space abnormalities in the cognition level is compatible with its function in the cognition status, and the negative correlation between basal ganglia dVRS volume fraction and MoCA/global cognition scores is consistent with the hypothesized detrimental effect of these structures on cognition in PD (higher dVRS burden leads to lower cognitive test scores, resulting in poorer performance) [4]. It should be mentioned that these results were not completely supported by other studies. A retrospective cohort by Fang et al. on 245 PD patients and 98 healthy control subjects showed that there was no significant association between cognitive domains (global cognition, memory, working memory, or executive function) and the MoCA score [12]. In addition to analytical experiments and surveys, there are some case reports in the literature reporting degrees of cognitive decline in PD patients with dVRS. Shoeibi and Litvan reported a patient with dVRS structures in the posterior fossa—some extending 1.5 cm in diameter—with mild executive function impairment described as difficulties with retrieval with normal encoding and decreased fluency (MoCA = 25 of 30) [15].

Not only because of the impaired clearance function but also due to the mass effect and potential tendency of developing multiple infarcts in the brain (importantly, in the frontal lobe), the dVRS is considered a potential risk factor for the development of worsened cognitive decline in PD patients [5–7]. In a study conducted by Zhang et al. on patients with lacunar infarcts, 89 patients with dVRS in basal ganglia and 178 controls with random distribution of vascular risk factors were studied, and the results showed that more severe dVRS (in terms of size and quantity) was compatible with more lacunar infarct lesions and more brain atrophy, which may present in the areas affecting the cognitive process [5]. They also reported brain atrophy (OR = 1.40; 95%CI 1.13, 1.73) and white‐matter lesions (OR = 1.88; 95% CI 1.24, 2.83) as independent risk factors for the presence of basal ganglia dVRS structures [5]. In contrast, Tu et al. found no significant within‐PD association: the number and location of enlarged perivascular spaces did not differ between PD patients with and without cognitive impairment (all *p* > 0.05), and the significant basal ganglia associations reported in that study were confined to the vascular parkinsonism group rather than the PD group [7]. This study therefore does not support a direct dVRS–cognition association in PD.

### 3.6. Effects on SNSB Score

The abovementioned correlations with the attendant correlation coefficients are described in more detail in a study conducted by Young Shin et al. where they analyzed 36 PD patients with intact cognition, 111 with mild cognitive impairment, and 7 with dementia, and their relation with hypertensive status [13]. They defined cognitive impairment as the score below one standard deviation in one of the domains (age, sex, and education‐matched norms) of the SNSB, and the results were as follows (Table 1) [13].

Hypertension (*β* = 0.138, SE = 0.148, *p* = 0.045) and supine hypertension (*β* = 0.167, SE = 0.266, *p* = 0.016) had a significant positive association with the basal ganglia dVRS. Furthermore, the basal ganglia dVRS (*β* = 0.440, SE = 0.079, *p* < 0.001) was associated with higher total white‐matter lesions; and higher total white‐matter lesion (*β* = 0.188, SE = 0.044 *p* = 0.047) was consequently related to impaired cognitive function. It should be mentioned that white‐matter dVRS structures were not significantly associated with cognitive status [13]. These hemodynamic findings can be interpreted within a glymphatic–vascular framework. Arterial wall motion is the principal driver of net CSF flow through perivascular spaces; chronic hypertension does not change vessel diameter but alters the pulse‐wave profile, increasing backflow and reducing net forward perivascular flow, thereby producing perivascular stasis [10]. The lenticulostriate arteries supplying the basal ganglia are particularly exposed to this effect because they branch directly from the middle cerebral artery without intervening damping arborization [9, 10]. The clustering of hypertension, basal ganglia dVRS, periventricular white‐matter lesions, and impaired cognition observed by Shin et al. [9] is therefore consistent with this mechanistic chain rather than four independent associations.

In addition, it is worth mentioning that the basal ganglia dVRS had positive association with both periventricular and deep white‐matter lesions, but only the former type of lesions had a significant correlation with poor cognitive status (*β* = 0.188, SE = 0.044, *p* = 0.047) [13].

### 3.7. Effects on Other (and Multiple) Cognitive Assessment Tools

Some studies assessed the effect of dVRS on multiple cognitive assessment tools, thus making a comparative view of the effect of these structures on each test. Lee and colleagues performed a study on 11 cases and reported that semiquantitative and quantitative dVRS scores had strong negative correlation with the Frontal Assessment Battery and Korean version of MoCA, and moderate correlation with Korean MMSE (Table 1) [20].

The abovementioned studies elucidated the effect of the relations between vascular risk factors and the development of dVRS structures, and the relation between these structures and clinical cognitive impairment; however, not all the studies support the relation between dVRS and cognitive impairment. In a study performed by Shen et al., it was noted that the VRS indexes (size and volume) were not significantly associated with scores in MoCA, MMSE, and PANDA [14]. This may reveal the fact that there is no exact and always‐present relation between dVRS structures and cognitive decline in PD patients.

Some PD case reports in the literature were reported with cognitive impairment accompanied by the dVRS; however, it is not clear if the mentioned poor cognition status is related to the dVRS. A PD patient with impaired visuospatial and memory function was reported in the literature with VRS structural abnormalities in the periventricular white‐matter and subcortical areas of frontoparietal, left temporal, and left occipital lobes [1]. In another case report, bilateral basal ganglia dVRS was accompanied by mild delayed memory in a PD patient [16].

### 3.8. SCS

When notably enlarged, the dVRS in the striatum is considered SCS, which is more common in elderly patients [8]. A study conducted on MRI reports of patients aged 40 or older with SCS (*n* = 27) presenting at the Mayo Clinic from 2000 to 2007 investigated if this condition may significantly affect the clinical presentation of the patients compared to the control patients without SCS (*n* = 52). The mentioned study used mild cognitive impairment, dementia of any type, dementia with psychosis or dream enactment, and history of neuropsychological testing as the cognitive status items for comparison between case and control groups; and results showed that the SCS cases were not significantly different from controls in terms of presenting with mild cognitive impairment (15.0% vs. 8.0%, *p* value = 0.44), dementia of any type (30.0% vs. 21.0%, *p* value = 0.40), dementia with psychosis or dream enactment (11.0% vs. 12.0%, *p* value = 0.99), and history of neuropsychological testing (26.0% vs. 19.0%, *p* value = 0.49) [8]. It should be mentioned that not all of the SCS patients in the mentioned study were PD patients; however, the two groups were not significantly different in terms of presenting parkinsonian symptoms as well (19.0% vs. 17.0%, *p* value = 0.90) [8]. Only 19% of the SCS cases (and 17% of controls) presented with parkinsonian symptoms [8]; the cohort was therefore a mixed elderly sample rather than a PD‐specific cohort. The null result for cognitive endpoints in this study should not be generalized to the PD‐specific cohorts reviewed earlier [4, 7, 13], in which dVRS, and basal ganglia dVRS, in particular, was associated with poorer cognitive performance (Table 1).

### 3.9. Summary of the Direct PD Evidence

Across the primary PD‐cognition studies, findings were inconsistent. Two studies reported significant negative associations between perivascular‐space burden and cognitive performance: Donahue et al. [4], in which basal ganglia and rostral‐middle‐frontal perivascular‐space volume fraction were negatively associated with global cognition and MoCA after adjustment, and Stagisa et al. [17], in which basal ganglia and centrum‐semiovale perivascular‐space counts correlated negatively with MoCA. One study was mixed and region‐dependent [18]. Centrum‐semiovale burden was associated with worse cognition, basal ganglia burden with motor rather than cognitive impairment. Two studies were null for global cognition and most domains (Fang et al. [12], with an isolated association limited to attention–processing speed; Shen et al. [14]). Tu et al. [7] were null within the PD group. One study described an indirect relationship in which basal ganglia perivascular‐space burden was linked to cognition only through mediation by white‐matter‐lesion burden rather than directly [13]. The three PD case reports [1, 15, 16] document co‐occurrence of dVRS and cognitive impairment but cannot establish an association. Overall, the direct PD evidence does not demonstrate a consistent dVRS–cognition association; this heterogeneity is concordant with a recent meta‐analysis reporting conflicting cognitive associations in PD glymphatic‐imaging studies [21].

### 3.10. A Discussion of This Review

This systematic review of 16 studies examined the reported relationships between dVRS and cognitive impairment in patients with PD. Across directly comparable PD cohorts, the association between basal ganglia dVRS and cognition was inconsistent: some studies reported significant negative associations with global cognition and MoCA [4, 17, 18], whereas others were null [7, 8, 14] or found only an indirect, white‐matter‐lesion‐mediated relationship [13]. The evidence is therefore best summarized as suggestive but not consistent. The supporting mechanisms proposed in the included studies (impaired glymphatic waste clearance, mechanical mass effect, and increased small‐vessel ischemic burden) may converge on the glymphatic–vascular interface, although this integrative model is hypothesis‐generating and is not established by the included PD studies. The mechanisms by which dVRS may contribute to cognitive decline in PD are best understood as plausible, partially supported pathways rather than established explanations, given that some of the supporting evidence derives from related but not directly comparable populations (vascular parkinsonism, lacunar‐stroke cohorts, mixed elderly cohorts with SCS).

### 3.11. Mechanistic Interpretation

Three non‐mutually exclusive mechanisms have been proposed. None has been directly validated against cognitive outcomes in a PD cohort within the included studies, and each draws partly on experimental or non‐PD evidence; they are therefore presented as possible explanations rather than an established mechanism. First, glymphatic clearance failure: arterial wall pulsations are the principal driver of net CSF flow through perivascular spaces [8]; reduced or impaired pulsatile flow leads to perivascular stasis and accumulation of misfolded α‐synuclein, a key substrate of PD pathology [3]. Second is mechanical mass effect. When perivascular spaces dilate to a giant or unusual degree, as in the included case reports [15, 16], they may compress and displace adjacent structures, potentially including cognition‐related white‐matter tracts (IFOF, ILF). It remains unclear, however, whether such mechanical effects contribute to the cognitive findings associated with routine MRI‐visible dVRS in PD, in which the spaces are typically small; this mechanism should be regarded as speculative for the common phenotype; this driver has been demonstrated primarily in rodent experimental models and has not been directly linked to cognitive outcomes in the included PD studies. Third is the increased small‐vessel ischemic burden. Severe basal ganglia dVRS is associated with concurrent lacunar infarcts, white‐matter hyperintensities, and brain atrophy in cohorts with PD or vascular parkinsonism [5–7], reflecting shared vascular risk and mechanism. Several of these associations derive from vascular parkinsonism or lacunar‐stroke cohorts [5–7] rather than from PD‐specific cognitive data. The clustering of hypertension, basal ganglia dVRS, periventricular white‐matter lesions, and impaired cognition observed by Shin et al. [13] is consistent with this mechanistic chain rather than four independent associations: chronic hypertension increases pulse‐wave amplitude and reduces damping at the lenticulostriate level, paradoxically reducing net perivascular flow and producing perivascular stasis [8].

### 3.12. Reasons for Interstudy Discrepancies

Not all studies reported significant dVRS–cognition associations. Shen et al. [14] found no significant association between PVS indices and MoCA, MMSE, or PANDA scores in early‐stage PD; Burnett et al. [8] found no significant difference in mild cognitive impairment between cases with SCS and controls. Several methodological factors plausibly explain this heterogeneity. Considering MRI field‐strength heterogeneity, the included studies used 1.5T–3.0T systems; visibility of small (< 1 mm) PVS is substantially lower at 1.5T than at 3.0T or higher field strengths, which may attenuate detected associations [22]. Absence of a single standardized dVRS rating method may also play a role. Visual ordinal scales (e.g., Potter et al. [23]), semiquantitative counts on a single axial slice, and automated volume‐fraction quantification each capture different aspects of perivascular burden and have differing interrater reliabilities [22, 23]. Heterogeneous PD severity at enrolment is also notable. Shen et al. enrolled early‐stage PD (Hoehn‐Yahr I‐II), in which cognitive change may be too modest to detect dVRS associations, whereas Tu et al. and Donahue et al. included a broader severity range [4, 7, 14]. Variable confounder adjustment may also affect. Some studies adjusted for age, sex, education, hypertension, and concurrent small‐vessel‐disease markers, while others adjusted for fewer covariates. Finally, it is important to note that the Burnett et al. cohort [8] was a mixed elderly population in which only 19% of cases (and 17% of controls) had parkinsonian symptoms; its null finding therefore should not be generalized to PD‐specific cohorts.

### 3.13. Limitations of the Evidence Base

Several limitations constrain the strength of the conclusions. Predominantly cross‐sectional designs preclude causal inference. It is not possible to determine whether dVRS contributes to cognitive decline, follows from a shared neurodegenerative or vascular process, or both. Heterogeneity of dVRS rating methods, MRI field strength, and cognitive instruments precludes formal meta‐analysis. The four included case reports provide low‐strength evidence, although they are useful for hypothesis generation. Also, several included studies had modest sample sizes and were performed at single centers, limiting external generalizability. Inconsistent confounder adjustment, particularly for age, hypertension, and concurrent small‐vessel‐disease burden, may produce residual confounding. Some of the supporting mechanistic evidence derives from related but not PD‐specific populations (vascular parkinsonism, lacunar‐stroke, mixed elderly cohorts), and inferences drawn from those cohorts to PD‐specific cognitive trajectories should be interpreted cautiously. Finally, the systematic review itself was limited to three databases and English‐language articles, and grey literature was not searched, raising the possibility of publication bias.

### 3.14. Future Directions

Five priorities emerge from the present synthesis [1]. Prospective longitudinal cohorts of PD patients with serial structural MRI and standardized cognitive batteries are needed to test whether dVRS predicts incident cognitive decline beyond baseline cognitive status and other small‐vessel‐disease markers [2]. Consensus on a single standardized dVRS rating method, ideally automated and validated against 7T MRI ground truth, is needed to enable cross‐study comparison and meta‐analysis [22, 23] [3]. Integration of dVRS burden with diffusion tensor image analysis along the perivascular space and other glymphatic‐imaging metrics may yield a more sensitive composite biomarker of glymphatic dysfunction in PD [9] [4]. Interventional studies (most plausibly trials of intensive blood‐pressure control or treatments targeting aquaporin‐4 function) could test whether modifying the underlying mechanisms slows dVRS progression and cognitive decline in PD [5]. Post‐mortem validation of MRI‐defined dVRS against histopathological perivascular‐space morphology and α‐synuclein burden would clarify the molecular substrates of MRI‐visible dVRS in PD.

## 4. Conclusion

In summary, this systematic review of 16 studies indicates that dilated perivascular spaces, particularly in the basal ganglia, have been associated with lower cognitive test scores in some studies of patients with PD, although the direct PD evidence is mixed, with several null or indirect findings. The MoCA was the most commonly used assessment tool for cognitive impairment in the included studies, with the MMSE, FAB, SNSB, and PANDA also used. Three proposed mechanisms (impaired glymphatic waste clearance (notably of α‐synuclein), mechanical mass effect, and a heightened tendency for small‐vessel ischemic injury) may converge on the glymphatic–vascular interface; however, this integrative account is hypothesis‐generating, has not been established by the included studies, and each mechanism should be regarded as a possible contributing pathway rather than a validated, unifying biological substrate. Overall, basal ganglia dVRS is associated with vascular risk factors (notably hypertension); its association with cognitive performance in PD is inconsistent across studies.

For clinicians and translational researchers, the present evidence is insufficient to establish basal ganglia (or centrum semiovale/cortical) dVRS as an independent cognitive marker in PD. No included study evaluated screening performance or incremental predictive value over established markers, and no formal certainty‐of‐evidence (e.g., GRADE) grading was undertaken; the association should therefore be regarded as a hypothesis for prospective testing rather than a validated biomarker. Basal ganglia dVRS therefore warrants further investigation as a candidate imaging biomarker for early cognitive screening in PD, ideally as part of a composite small‐vessel‐disease score; longitudinal validation against incident cognitive decline is still required before clinical adoption.

## Funding

No funding sources were available for this project.

## Disclosure

All authors agree to be accountable for the research presented.

## Ethics Statement

We confirm that we have read the Journal’s position on issues involved in ethical publication and affirm that this work is consistent with those guidelines. The present paper is also approved by ethics committee of Shahid Beheshti University of Medical Sciences. The authors confirm that the approval of an institutional review board and the patient consent was not required for this work.

## Conflicts of Interest

The authors declare no conflicts of interest.

## Supporting Information

Additional supporting information can be found online in the Supporting Information section.

## Supporting information


**Supporting Information 1** Supporting Material S1. (a) Risk of bias—JBI Analytical Cross‐Sectional Studies; (b) Risk of bias—JBI Case Series; and (c) Risk of bias—JBI Case Reports.

**Supporting Information 2** Supporting Material S1. Database‐specific search strategies, including entry terms and Boolean structure in each database, search date, and number of results.

## Data Availability

This systematic review does not contain any data deposited in a data depository.

## References

[1] Kim D. G. , Oh S. H. , and Kim O. J. , A Case of Disseminated Polycystic Dilated Perivascular Spaces Presenting With Dementia and Parkinsonism, Journal of Clinical Neurology. (2007) 3, no. 2, 96–100, 10.3988/jcn.2007.3.2.96. PMC2686866 19513299

[2] Scollato A. , Gallina P. , and Di Lorenzo N. , Cerebrospinal Fluid Diversion in Patients With Enlarged Virchow-Robin Spaces Without Ventriculomegaly, Acta Neurol Scand. (2016) 133, no. 1, 75–80, https://www.scopus.com/inward/record.uri?eid=2-s2.0-84954365237%26doi=10.1111%2Fane.12419%26partnerID=40%26md5=a672366b35a47817365424eaca805a4a, 10.1111/ane.12419. 25932744

[3] Buccellato F. R. , D’Anca M. , Serpente M. , Arighi A. , and Galimberti D. , The Role of Glymphatic System in Alzheimer’s and Parkinson’s Disease Pathogenesis, Biomedicines. (2022) 10, no. 9, 10.3390/biomedicines10092261. PMC9496080 36140362

[4] Donahue E. K. , Foreman R. P. , Duran J. J. et al., Increased Perivascular Space Volume in White Matter and Basal Ganglia is Associated With Cognition in Parkinson’s Disease, Brain Imaging Behav. (2024) 18, no. 1, 57–65, 10.1007/s11682-023-00811-4. PMC10844402 37855955

[5] Zhang X. Y. , Ding L. L. , Yang L. et al., Brain Atrophy Correlates With Severe Enlarged Perivascular Spaces in Basal Ganglia Among Lacunar Stroke Patients, Public Library of Science. (2016) 11, no. 2, 10.1371/journal.pone.0149593. PMC4764761 26900696

[6] Donahue E. K. , Murdos A. , Jakowec M. W. et al., Global and Regional Changes in Perivascular Space in Idiopathic and Familial Parkinson’s Disease, Movement Disorders. (2021) 36, no. 5, 1126–1136, 10.1002/mds.28473. PMC8127386 33470460

[7] Tu Y. , Zhuo W. Y. , Peng J. W. et al., The Correlation Between Enlarged Perivascular Spaces and Cognitive Impairment in Parkinson’s Disease and Vascular Parkinsonism, BioMed Central Neurology. (2022) 22, no. 1, 10.1186/s12883-022-02819-7. PMC9336003 35906550

[8] Burnett M. S. , Witte R. J. , and Ahlskog J. E. , Swiss Cheese Striatum Clinical Implications, Journal of the American Medical Association Neurology. (2014) 71, no. 6, 735–741, https://www.scopus.com/inward/record.uri?eid=2-s2.0-84902196079%26doi=10.1001%2Fjamaneurol.2014.286%26partnerID=40%26md5=c1508c4be34c3b669ccd100ba72e16a6, 10.1001/jamaneurol.2014.286. 24733660

[9] Shen T. , Yue Y. , Ba F. et al., Diffusion Along Perivascular Spaces as Marker for Impairment of Glymphatic System in Parkinson’s Disease, NPJ Parkinson’s Diseas. (2022) 8, no. 1, 10.1038/s41531-022-00437-1. PMC9772196 36543809

[10] Mestre H. , Hablitz L. M. , Xavier A. L. R. et al., Aquaporin-4-Dependent Glymphatic Solute Transport in the Rodent Brain, eLife. (2018) 7, 10.7554/elife.40070. PMC6307855 30561329

[11] Takkouche B. and Norman G. , PRISMA Statement, Epidemiology. (2011) 22, no. 1. 10.1097/EDE.0b013e3181fe7825 21150360

[12] Fang Y. , Gu L. Y. , Tian J. et al., MRI-Visible Perivascular Spaces are Associated With Cerebrospinal Fluid Biomarkers in Parkinson’s Disease, Aging-US. (2020) 12, no. 24, 25805–25818, 10.18632/aging.104200. PMC7803484 33234732

[13] Shin N. Y. , Park Y. W. , Yoo S. W. et al., Adverse Effects of Hypertension, Supine Hypertension, and Perivascular Space on Cognition and Motor Function in PD, NPJ Parkinson’s Disease. (2021) 7, no. 1, 10.1038/s41531-021-00214-6. PMC8355129 34376695

[14] Shen T. , Yue Y. M. , Zhao S. et al., The Role of Brain Perivascular Space Burden in Early-Stage Parkinson’s Disease, NPJ Parkinson’s Disease. (2021) 7, no. 1, 10.1038/s41531-021-00155-0. PMC7864928 33547311

[15] Shoeibi A. and Litvan I. , Levodopa-Responsive Parkinsonism Associated With Giant Virchow-Robin Spaces: A Case Report, Movement Disorders Clinical Practice. (2017) 4, no. 4, 619–622, https://www.scopus.com/inward/record.uri?eid=2-s2.0-85067374034%26doi=10.1002%2Fmdc3.12484%26partnerID=40%26md5=40c38b7e23a128fe7fc1539dd945f1c1, 10.1002/mdc3.12484. PMC6353514 30713969

[16] López Fernández M. , Fraga Bau A. , Volkmer García C. M. , and Canneti Heredia B. , Virchow-Robin Spaces: A Cause of Parkinsonism?, Neurologia. (2016) 31, no. 7, 493–494, https://www.scopus.com/inward/record.uri?eid=2-s2.0-84923455640%26doi=10.1016%2Fj.nrl.2015.01.002%26partnerID=40%26md5=4dcae463532ccb3fc345c0cc067aa5d8, 10.1016/j.nrleng.2015.01.001. 25728953

[17] Stagisa E. , Silovs A. , Skuburs G. K. et al., Quantitative and Qualitative MRI Assessment of Perivascular Spaces in Parkinson’s Disease Patients, Medicina (B Aires). (2026) 62, no. 4, 10.3390/medicina62040613. PMC13117765 42075485

[18] Li Q. , Bai X. , Zhang S. et al., The Association of Enlarged Perivascular Space With Cerebrospinal Fluid GFAP in Early Parkinson’s Disease, Academic Radiology. (2025) 33. 10.1016/j.acra.2025.11.008 41309400

[19] Alonso F. , Zsigmond P. , and Wårdell K. , Influence of Virchow-Robin Spaces on the Electric Field Distribution in Subthalamic Nucleus Deep Brain Stimulation, Clinical Neurology and Neurosurgery. (2021) 204, https://www.scopus.com/inward/record.uri?eid=2-s2.0-85103676774%26doi=10.1016%2Fj.clineuro.2021.106596%26partnerID=40%26md5=ca77a03e406abe3dd4ba0984d85c9f24. 10.1016/j.clineuro.2021.106596 33813373

[20] Lee D. , Hong I. K. , and Ahn T. B. , Dilated Virchow-Robin Space and Dopamine Transporter Imaging in the Striatum of Patients With Parkinsonism, Canadian Journal of Neurological Sciences. (2015) 42, no. 4, 248–254, https://www.scopus.com/inward/record.uri?eid=2-s2.0-85018223752%26doi=10.1017%2Fcjn.2015.43%26partnerID=40%26md5=890c30bd424447741b9824ecaffcbb80, 10.1017/cjn.2015.43. 26153041

[21] Rajai F. S. , Mohammadi I. , Aghajanian S. et al., Glymphatic Dysfunction in Parkinson’s Disease: A Systematic Review and Meta‐Analysis of Neuroimaging Studies, Movement Disorders Clinical Practice. (2026) 13, no. 2, 331–343, 10.1002/mdc3.70296. PMC12911525 40817751

[22] Duering M. , Biessels G. J. , Brodtmann A. et al., Neuroimaging Standards for Research Into Small Vessel Disease—Advances Since 2013, Lancet Neurol. (2023) 22, no. 7, 602–618, 10.1016/s1474-4422(23)00131-x. 37236211

[23] Potter G. M. , Chappell F. M. , Morris Z. , and Wardlaw J. M. , Cerebral Perivascular Spaces Visible on Magnetic Resonance Imaging: Development of a Qualitative Rating Scale and Its Observer Reliability, Cerebrovascular Diseases. (2015) 39, no. 3–4, 224–231, 10.1159/000375153. PMC4386144 25823458

