# Supplementary material for: Dilated Virchow–Robin Space and Cognitive Impairment in People With Parkinson’s Disease: A Systematic Review
Source: Radiol Res Pract. 2026 Nov 6;2026:6959099. doi: 10.1155/rrp/6959099 (PMC13743799; doi:10.1155/rrp/6959099)
Supplement: Supplementary file 1 [file RRP-2026-6959099-s001.docx]

**Supplementary Table S1a. Risk of bias - JBI Analytical Cross-Sectional Studies**

Two reviewers; verdicts: Y = Yes, N = No, U = Unclear, NA = Not applicable. Item key below the table.

| **Study (ref)** | **Q1** | **Q2** | **Q3** | **Q4** | **Q5** | **Q6** | **Q7** | **Q8** | **Overall** |
| --- | --- | --- | --- | --- | --- | --- | --- | --- | --- |
| Donahue et al., 2024 | **Y** | **Y** | **Y** | **Y** | **Y** | **Y** | **Y** | **Y** | **Low** |
| Donahue et al., 2021 | **Y** | **Y** | **Y** | **Y** | **Y** | **Y** | **NA*** | **Y** | **Low–moderate** |
| Zhang et al., 2016 | **Y** | **Y** | **U** | **Y** | **Y** | **Y** | **NA*** | **Y** | **Moderate** |
| Tu et al., 2022 | **Y** | **Y** | **U** | **Y** | **Y** | **Y** | **Y** | **Y** | **Low–moderate** |
| Burnett et al., 2014 | **Y** | **Y** | **Y** | **U** | **Y** | **Y** | **U** | **Y** | **Moderate** |
| Fang et al., 2020 | **Y** | **Y** | **Y** | **Y** | **Y** | **Y** | **Y** | **Y** | **Low** |
| Shin et al., 2021 | **Y** | **Y** | **Y** | **Y** | **Y** | **Y** | **Y** | **Y** | **Low** |
| Shen et al., 2021 | **Y** | **Y** | **Y** | **Y** | **Y** | **Y** | **Y** | **Y** | **Low** |
| Stagisa et al., 2026 | **Y** | **Y** | **Y** | **Y** | **Y** | **Y** | **Y** | **Y** | **Low** |
| Li et al., 2025 | **Y** | **Y** | **Y** | **U** | **Y** | **Y** | **Y** | **Y** | **Low** |

**Item key**

Q1 Inclusion criteria clearly defined

Q2 Subjects & setting described in detail

Q3 Exposure (dVRS) measured validly & reliably

Q4 Objective, standard criteria for the condition (PD)

Q5 Confounding factors identified

Q6 Strategies to deal with confounding stated

Q7 Outcomes (cognition) measured validly & reliably

Q8 Appropriate statistical analysis

**Notes**

• * Donahue 2021 and Zhang 2016 did not assess cognition as an outcome (Donahue 2021 compared PVS burden between groups; Zhang 2016 examined risk factors for BG-EPVS in lacunar-stroke patients) - Q7 marked NA for the cognitive outcome.

• Tu 2022 and Zhang 2016 did not report inter-rater reliability for dVRS rating (Q3 = Unclear).

• Burnett 2014 ascertained cognition retrospectively from medical records (Q7 = Unclear) in a mixed elderly population only ~19% of whom had parkinsonism (Q4 = Unclear for PD-specific applicability).

**Supplementary Table S1b. Risk of bias - JBI Case Series**

| **Study (ref)** | **Q1** | **Q2** | **Q3** | **Q4** | **Q5** | **Q6** | **Q7** | **Q8** | **Q9** | **Q10** | **Overall** |
| --- | --- | --- | --- | --- | --- | --- | --- | --- | --- | --- | --- |
| Lee et al., 2015 | **Y** | **Y** | **Y** | **Y** | **Y** | **Y** | **Y** | **Y** | **Y** | **U** | **Moderate** |
| Scollato et al., 2016 | **Y** | **Y** | **Y** | **U** | **Y** | **Y** | **Y** | **Y** | **Y** | **NA** | **Moderate** |
| Alonso et al., 2021 | **U** | **Y** | **Y** | **U** | **U** | **Y** | **Y** | **Y** | **Y** | **NA** | **Moderate–high** |

**Item key**

Q1 Clear inclusion criteria

Q2 Condition measured reliably for all

Q3 Valid methods to identify the condition

Q4 Consecutive inclusion

Q5 Complete inclusion

Q6 Demographics clearly reported

Q7 Clinical information clearly reported

Q8 Outcomes/follow-up clearly reported

Q9 Presenting site(s) demographics reported

Q10 Appropriate statistical analysis

**Notes**

• Lee 2015 used unadjusted Spearman correlations in a small (n=11), clinically heterogeneous sample (only 4 PD); Q10 = Unclear given absence of confounding adjustment.

• Scollato 2016 and Alonso 2021 are descriptive/biomechanical; formal inferential statistics were not the aim (Q10 = NA). Alonso 2021 included a selected DBS subgroup with VRS (Q1/Q4/Q5 = Unclear) and did not assess cognition.

**Supplementary Table S1c. Risk of bias - JBI Case Reports**

Two reviewers; verdicts: Y = Yes, N = No, U = Unclear, NA = Not applicable. Item key below the table.

| **Study (ref)** | **Q1** | **Q2** | **Q3** | **Q4** | **Q5** | **Q6** | **Q7** | **Q8** | **Overall** |
| --- | --- | --- | --- | --- | --- | --- | --- | --- | --- |
| Shoeibi & Litvan, 2017 (19) | **Y** | **Y** | **Y** | **Y** | **Y** | **Y** | **U** | **Y** | **Low–moderate** |
| López Fernández et al., 2016 (20) | **Y** | **Y** | **Y** | **Y** | **U** | **U** | **U** | **Y** | **Moderate** |
| Kim et al., 2007 (1) | **Y** | **Y** | **Y** | **Y** | **Y** | **Y** | **U** | **Y** | **Low–moderate** |

**Item key**

Q1 Patient's demographic characteristics clearly described

Q2 Patient's history clearly described and presented as a timeline

Q3 Current clinical condition on presentation clearly described

Q4 Diagnostic tests / assessment methods and their results clearly described

Q5 Intervention(s) or treatment procedure(s) clearly described

Q6 Post-intervention clinical condition clearly described

Q7 Adverse events (harms) or unanticipated events identified and described

Q8 Takeaway lessons provided

**Notes**

• None of the three reports is a quantitative study; none contributes an effect estimate to the synthesis. All three are carried in Group 1 of Table 1 as descriptive evidence of dVRS-cognition co-occurrence only, so their appraisal bears on descriptive completeness rather than on risk of bias in an estimated association.

• Instrument: JBI Critical Appraisal Checklist for Case Reports (8 items), applied because all three articles report a single patient. Studies reporting more than one patient are appraised in Supplementary Table S1b with the JBI Case Series checklist.
