# Supplementary material for: Dilated Virchow–Robin Space and Cognitive Impairment in People With Parkinson’s Disease: A Systematic Review
Source: Radiol Res Pract. 2026 Nov 6;2026:6959099. doi: 10.1155/rrp/6959099 (PMC13743799; doi:10.1155/rrp/6959099)
Supplement: Supplementary file 2 [file RRP-2026-6959099-s002.docx]

**Supplementary Table S2. Database-specific search strategies**

*All three databases were searched from 01 January 2000 to 01 July 2026, using the entry terms and Boolean structure given in the Methods, adapted below to each database's own field-tag syntax.*

| **Database** | **Search string (adapted to database syntax)** | **Date range** | **Records retrieved** |
| --- | --- | --- | --- |
| PubMed | (("virchow robin*"[Title/Abstract] OR "virchow-robin"[Title/Abstract])  AND  (parkinson*[Title/Abstract]))  AND  (cognit*[Title/Abstract] OR metacognit*[Title/Abstract] OR awareness[Title/Abstract] OR comprehens*[Title/Abstract] OR conscious*[Title/Abstract] OR intuit*[Title/Abstract] OR imagin*[Title/Abstract]) | 2000-01-01 to 2026-07-01 | 12 |
| Scopus | (TITLE-ABS-KEY(“virchow robin*” OR “virchow-robin”)  AND  TITLE-ABS-KEY(parkinson*))  AND  TITLE-ABS-KEY(cognit* OR metacognit* OR awareness OR comprehens* OR conscious* OR intuit* OR imagin*) | 2000-01-01 to 2026-07-01 | 28 |
| Web of Science | TS=((“virchow robin*” OR “virchow-robin”) AND parkinson*)  AND  TS=(cognit* OR metacognit* OR awareness OR comprehens* OR conscious* OR intuit* OR imagin*) | 2000-01-01 to 2026-07-01 | 31 |
